# Salvianolic Acid B Inhibits Hydrogen Peroxide-Induced Endothelial Cell Apoptosis through Regulating PI3K/Akt Signaling

**DOI:** 10.1371/journal.pone.0001321

**Published:** 2007-12-19

**Authors:** Chen-Li Liu, Li-Xia Xie, Min Li, Siva Sundara Kumar Durairajan, Shinya Goto, Jian-Dong Huang

**Affiliations:** 1 School of Chinese Medicine, Hong Kong Baptist University, Hong Kong, China; 2 Department of Biochemistry, Li Ka Shing Faculty of Medicine, The University of Hong Kong, Hong Kong, China; 3 Department of Medicine, School of Medicine, Tokai University, Hiratsuka, Kanagawa, Japan; National Cancer Institute at Frederick, United States of America

## Abstract

**Background:**

Salvianolic acid B (Sal B) is one of the most bioactive components of *Salvia miltiorrhiza*, a traditional Chinese herbal medicine that has been commonly used for prevention and treatment of cerebrovascular disorders. However, the mechanism responsible for such protective effects remains largely unknown. It has been considered that cerebral endothelium apoptosis caused by reactive oxygen species including hydrogen peroxide (H_2_O_2_) is implicated in the pathogenesis of cerebrovascular disorders.

**Methodology and Principal Findings:**

By examining the effect of Sal B on H_2_O_2_-induced apoptosis in rat cerebral microvascular endothelial cells (rCMECs), we found that Sal B pretreatment significantly attenuated H_2_O_2_-induced apoptosis in rCMECs. We next examined the signaling cascade(s) involved in Sal B-mediated anti-apoptotic effects. We showed that H_2_O_2_ induces rCMECs apoptosis mainly through the PI3K/ERK pathway, since a PI3K inhibitor (LY294002) blocked ERK activation caused by H_2_O_2 _and a specific inhibitor of MEK (U0126) protected cells from apoptosis. On the other hand, blockage of the PI3K/Akt pathway abrogated the protective effect conferred by Sal B and potentated H_2_O_2_-induced apoptosis, suggesting that Sal B prevents H_2_O_2_-induced apoptosis predominantly through the PI3K/Akt (upstream of ERK) pathway.

**Significance:**

Our findings provide the first evidence that H_2_O_2_ induces rCMECs apoptosis via the PI3K/MEK/ERK pathway and that Sal B protects rCMECs against H_2_O_2_-induced apoptosis through the PI3K/Akt/Raf/MEK/ERK pathway.

## Introduction

Apoptosis is a process of programmed cell death in which defective and harmful cells are eliminated from a multicellular organism so as to maintain its homeostasis. Dysregulation of apoptotic signalling leads to pathological conditions, such as carcinoma (no apoptosis) and ischemia (enhanced apoptosis) [1]. Cerebral microvascular endothelial cells (CMECs) and intercellular tight junctions constitute the basic structure of the blood-brain barrier (BBB) which is responsible for regulating the trafficking of cells, substrates, and other molecules into the brain [2]. Apoptosis of CMECs may destroy the BBB and expose smooth muscle cells to neurotransmitters, toxins, and other vasoactive agents in the blood stream. Notably, CMECs apoptosis may lead to neuronal injury through the loss of BBB integrity and permit the extravasations of vascular inflammatory cells and proteins that are toxic to neurons [3]. Hence, CMECs apoptosis is considered to be partially responsible for the pathogenesis of various neurodisorders, such as cerebral ischemia, cerebral apoplexy, and Alzheimer's disease [4], [5].

It has been demonstrated that reactive oxygen species (ROS) are involved in the apoptosis of CMECs [6]. Production of high quantities of ROS within the vasculature occurs in a wide array of pathological events [7]. The excessive accumulation of ROS results in oxidative stress, which is known to induce cell death in a wide variety of cell types by modulating a series of intracellular signaling pathways [8]. Among these pathways, the activation of mitogen-activated protein kinases (MAPKs) and phosphatidylinositol-3-kinase (PI3K)/Akt pathways are known to play major roles in cell growth, survival, differentiation and apoptosis responses [9]. ROS that are particularly responsible for oxidative stress include hydrogen peroxide (H_2_O_2_), superoxide anions, and hydroxyl radicals. Among them, H_2_O_2_, the major source of endogenous ROS [10], is generated during hypoxia and ischemia-reperfusion injury [11], and has been extensively used to induce oxidative stress in *in vitro* models [7], [12].

The dried root of *Salvia miltiorrhiza* Bunge (Danshen) is a popular traditional Chinese medicine and has been widely used in both Asian and Western countries for the treatment of various diseases including cerebrovascular diseases, coronary artery diseases, and myocardial infarction [13], [14]. Salvianolic acid B (Sal B) is the most abundant and bioactive component of salvianolic acid in Danshen [15]. Extensive pharmacological studies have been carried out on this compound. It was shown that Sal B prevented ischaemia/reperfusion-induced rat brain injury by reducing lipid peroxidation, scavenging free radicals and improving energy metabolism [16]. In cerebral ischemia rats, Sal B reduced learning and memory dysfunctions induced by ischemia [17]. Moreover, salvianolic acids, including Sal B, were shown to improve regional cerebral blood flow in the ischemic hemisphere and inhibit platelet aggregation in rats [18]. More recently, Sal B was reported to be capable of improving the recovery of motor function after cerebral ischemia in rats [19]. At present, the molecular mechanisms responsible for the reported beneficial cerebrovascular effects of Sal B are relatively less studied. Considering the significance of oxidative stress-related cerebral vascular apoptosis, the present study was undertaken to examine the protective effects of Sal B on ROS (represented by H_2_O_2_)-induced rat cerebral microvascular endothelial apoptosis. We provide evidence that the anti-apoptotic effects of Sal B are at least in part mediated by altering the PI3K/Akt/Raf/MEK/ERK signaling pathway.

## Results

### Effects of Sal B on H_2_O_2_-induced apoptosis in rCMECs

**Figure 1:**
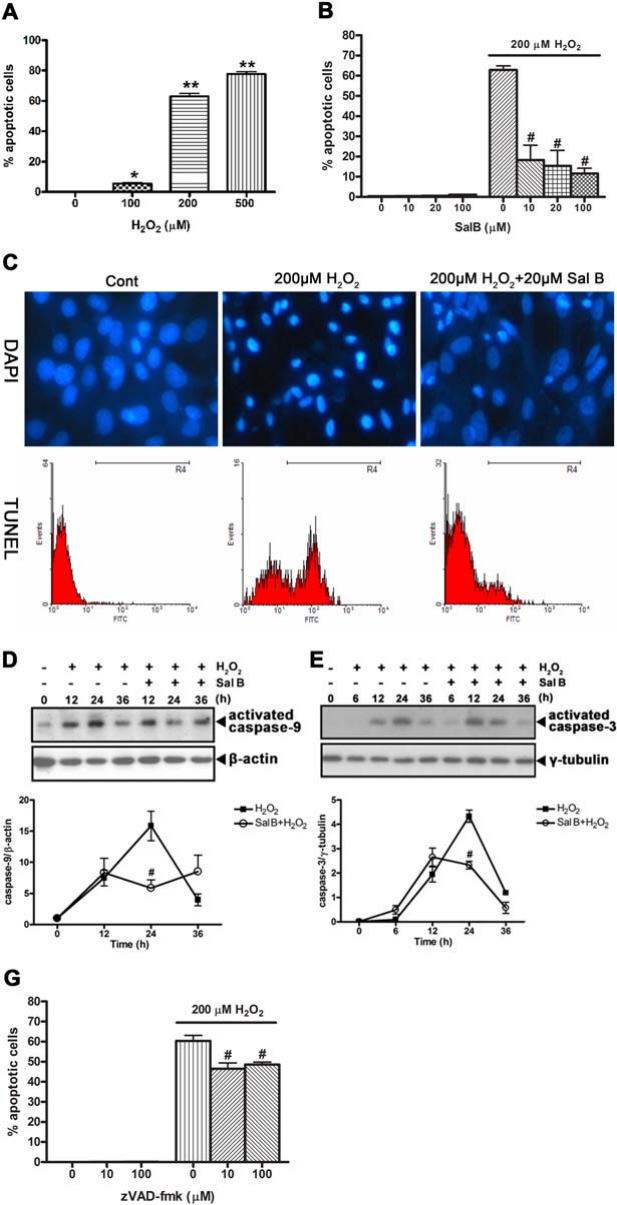
Inhibition of H_2_O_2_-induced rCMECs apoptosis by Sal B. (A) Apoptosis was induced in rCMECs with 0∼500 µM H_2_O_2_ for 12 h and determined by TUNEL assay. (B) rCMECs were pretreated with Sal B (0∼100 µM) for 30 min, and then coincubated with or without 200 µM H_2_O_2_ for 12 h, followed by apoptosis measurement using TUNEL assay. (C) rCMECs were analyzed by DAPI staining and TUNEL assay after a 12-h exposure to H_2_O_2_ with or without Sal B pretreatment. (D) Time course of caspase-9 activation in rCMECs incubated with H_2_O_2_ (200 µM) alone or with H_2_O_2_ (200 µM) and Sal B (20 µM). (E) Time course of caspase-3 activation in rCMECs incubated with H_2_O_2_ (200 µM) alone or with H_2_O_2_ (200 µM) and Sal B (20 µM). Immunoblotting were carried out on cell lysate proteins from control cells or rCMECs pretreated with Sal B for 1 h and then exposed to H_2_O_2_ for the indicated times. (G) rCMECs were pretreated with zVAD-fmk (0∼100 µM) for 60 min, and then coincubated with or without 200 µM H_2_O_2_ for 12 h, followed by apoptosis measurement using TUNEL assay. *P<0.05; **P<0.01 versus control, ^#^, *P*<0.05 versus H_2_O_2_ alone. Data are representative of three independent experiments.

We first measured H_2_O_2_-induced apoptosis in rCMECs using the TUNEL assay. As shown in Fig. 1A, the percentage of apoptotic (TUNEL-positive) cells increases dose-dependently with concentrations of H_2_O_2_ ranging from 100 to 500 µM for 12 h. In addition, we also evaluated nuclear condensation, which is characteristic for apoptotic cell death, using DAPI staining. To evaluate the effect of Sal B, cells were first pretreated with various concentrations of Sal B (from 10 to 100 µM), followed by treatment with H_2_O_2_ (200 µM for 12 h) and apoptosis was then quantified by TUNEL assay (Fig. 1B, 1C) and DAPI staining. Unstressed cells showed no signs of morpohological nuclear damage or chromatin condensation, which distinguished them from the stressed, H_2_O_2_-treated cells. The morphology of cells incubated with both H_2_O_2_ and Sal B was comparable to that of unstressed cells. To further verify the effect of Sal B on apoptosis induced by H_2_O_2_, TUNEL assays were performed. The results show that pretreatment with Sal B dose-dependently reduced H_2_O_2_-induced apoptosis (Fig. 1B).

We next examined caspase-9 and -3 activation in H_2_O_2_-stimulated endothelial cells. Western blotting analysis revealed that amounts of cleaved caspase-9 and -3 in H_2_O_2_-stimulated rCMECs were maximal at 24 h and returned to near basal concentrations at 36 h (Fig. 1D, E). However, these effects of H_2_O_2_ were attenuated by Sal B. Preincubation of cells with Sal B decreased the amounts of cleaved caspase-9 (Fig. 1D) and -3 (Fig. 1E), and also shortened the duration of their activation in response to exposure to H_2_O_2_. These data imply that Sal B may block the caspase-9 and -3 mediated apoptotic signaling pathways by acting on some upstream target(s). Given that activations of caspase-9 and -3 were still observed when Sal B significantly suppressed H_2_O_2_-induced apoptosis in the first 12 h, we sought to reveal the involvement of caspase. zVAD-fmk, a pan-caspase-inhibitor [20], was employed to examine its ability to prevent apoptosis by H_2_O_2_. The data shown in Fig. 1G demonstrates that zVAD-fmk only slightly reduced the apoptotic percentage after exposure to H_2_O_2_, implying that the majority of rCMECs may undergo caspase-independent apoptosis when exposed to 200 µM H_2_O_2_.

### Effects of Sal B on MEK/ERK signaling

**Figure 2:**
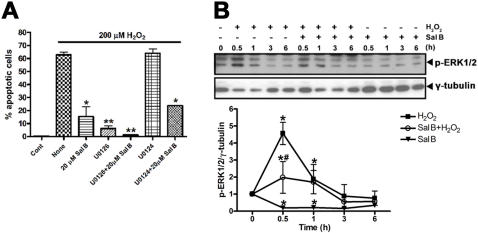
Effects of Sal B on MEK/ERK signaling. (A) Effects of Sal B, MEK inhibition, or their combination on H_2_O_2_-induced apoptosis. rCMECs were incubated with U0126 (10 µM) or U0124 (10 µM) for 1 h and then exposed to H_2_O_2_ in the presence or absence of Sal B pretreatment for 12 h. *P<0.05; **P<0.01 versus H_2_O_2_ alone. Data are representative of three independent experiments. (B) Time course of phosphorylated ERK1/2 expression in rCMECs incubated with H_2_O_2_ (200 µM) alone, or with H_2_O_2_ (200 µM) and Sal B (20 µM), or with Sal B (20 µM) alone. Immunoblotting were carried out on cell lysate proteins from control cells or rCMECs pretreated with Sal B for 1 h and then exposed to H_2_O_2_ for the indicated times. The representative Western blots and the quantitative analysis of protein expression (in relative protein density units). *P<0.05 versus control, ^#^, *P*<0.05 versus H_2_O_2_ alone. Data are representative of three independent experiments.

To investigate the molecular mechanism by which Sal B exerts its anti-apoptotic effects, the activation of MAPK was examined. An increasing body of evidence has shown that H_2_O_2_ stimulation increases extracellular signal-regulated kinase (ERK) activation and concomitant apoptosis [21], [22]. We performed the apoptosis analysis using U0126, a specific inhibitor of ERK upstream kinase MEK [23]. The increase of TUNEL-positive cells stimulated by H_2_O_2_ was significantly inhibited by U0126, but not by its inactive analogue U0124 [23] (Fig. 2A). These results indicated that H_2_O_2_-induced rCMECs apoptosis, which was attenuated by Sal B, was mediated through the MEK/ERK signaling pathway. Therefore, we wondered what the action of Sal B on the modulation of ERK activation in rCMECs is and whether the anti-apoptotic effect of Sal B is mediated through ERK. We thus analyzed ERK activation by Western blotting analysis with phospho-ERK-specific antibody. The results showed that amounts of phosphorylated ERK in H_2_O_2_-stimulated cells peaked at 30 min, that they returned to near basal concentrations after 3 h, but that pretreatment with Sal B resulted in a marked inhibition of these cellular responses, and that incubation of rCMECs with Sal B alone significantly reduced basal ERK phosphorylation (Fig. 2B).

### Role of PI3K signaling

**Figure 3:**
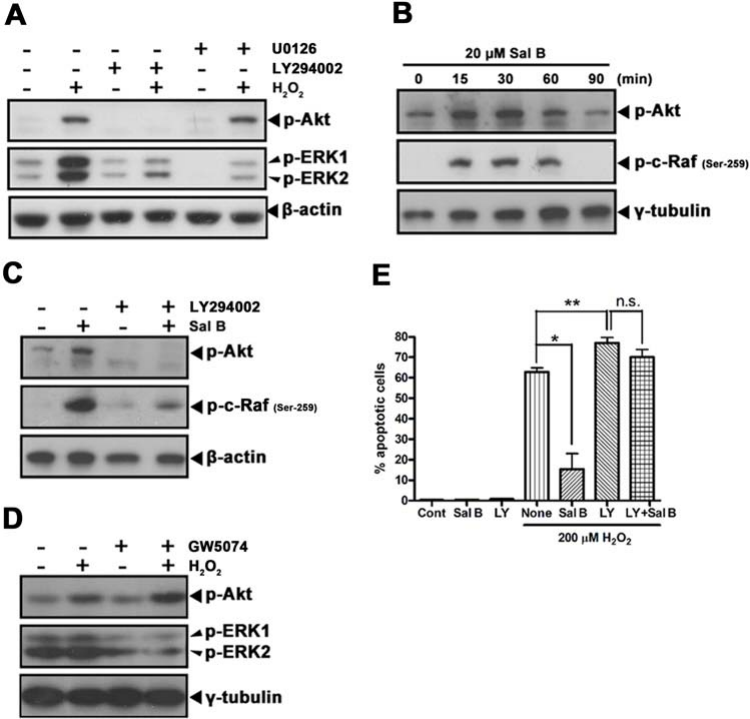
Role of PI3K signaling. (A) Effects of PI3K or MEK inhibition on phosphorylated Akt (Ser-473) and phosphorylated ERK1/2 expression in the presence or absence of H_2_O_2_ (200 µM). rCMECs were incubated with LY294002 (50 µM) or U0126 (10 µM) for 1 h and then exposed to H_2_O_2_ for 30 min. Blot shown is representative of at least three independent experiments. (B) Phosphorylated Akt (Ser-473) and phosphorylated c-Raf (Ser-259) expressions in rCMECs incubated with Sal B (20 µM) for the indicated times. (C) Effect of PI3K inhibition on phosphorylated Akt (Ser-473) and phosphorylated c-Raf (Ser-259) expressions induced by Sal B. rCMECs were incubated with LY294002 (50 µM) and/or Sal B (20 µM) for 1 h. (D) Effect of c-Raf inhibition on phosphorylated Akt (Ser-473) and phosphorylated ERK1/2 activation induced by H_2_O_2_. rCMECs were incubated with GW5074 (5 µM) and/or H_2_O_2_ (200 µM) for 1 h. (E) Effect of PI3K inhibition on H_2_O_2_-induced apoptosis in the presence or absence of Sal B (20 µM). rCMECs were incubated with LY294002 (50 µM) and/or Sal B (20 µM) for 1 h and then exposed to H_2_O_2_ for 12 h. LY, indicates LY294002. S, indicates Sal B. *P<0.05; **P<0.01 versus H_2_O_2_ alone; n.s., not significant. Data are representative of three independent experiments.

We next examined the effect of Akt inhibition on ERK phosphorylation in rCMECs exposed to H_2_O_2_. Treatment with LY294002, a specific inhibitor of Akt upstream kinase PI3K [24], resulted in the blockage of H_2_O_2_-induced ERK phosphorylation, as well as basal and H_2_O_2_-induced Akt phosphorylation. The basal level of ERK phosphorylation was also diminished (Fig. 3A). In the presence of U0126, basal and H_2_O_2_-induced ERK phosphorylation were blocked. However, U0126 had no effect on either basal or H_2_O_2_-induced Akt phosphorylation (Fig. 3A). These data clearly illustrate that PI3K acts upstream of ERK in the H_2_O_2_-induced signaling cascade. Previous studies have shown that Akt inhibited activation of the MEK/ERK signaling pathway by phosphorylating c-Raf at residue Ser-259 [25]. To investigate whether in rCMECs Sal B inhibited H_2_O_2_-induced MEK/ERK activation through Akt, we therefore evaluated the effect of Sal B on Akt activation. Results showed that the phosphorylation of Akt peaked at 15 min in the cells incubated with Sal B alone, and then returned to basal level over 60 min (Fig. 3B). An elevated level of phosphorylated c-Raf at Ser-259 was also triggered by Sal B alone (Fig. 3B). Furthermore, LY294002 treatment completely blocked expressions of phosphorylated Akt (Ser-473) and c-Raf (Ser-259) induced by Sal B (Fig. 3C). This indicates PI3K is required for Sal B-induced Akt activation and c-Raf deactivation. Since c-Raf is known to lie downstream of Akt, and upstream of ERK [25], [26], we then sought to confirm that this was also the case in rCMECs. GW5074, a selective inhibitor of c-Raf, inhibits the Raf/MEK/ERK cascade in *in vitro* assays by 90% at 5 µM [27]. Treatment with GW5074 had no effect on either basal or H_2_O_2_-induced Akt phosphorylation (Fig. 3D), but blocked H_2_O_2_-induced ERK phosphorylation (Fig. 3D). To further determine if the anti-apoptotic effects of Sal B were due to its effect on Akt, rCMECs were incubated with LY294002, with and without Sal B prior to H_2_O_2_ treatment. Inhibition of PI3K completely ablated the anti-apoptotic effect of Sal B, as well as H_2_O_2_-induced apoptosis was potentiated (Fig. 3E). Thus, these results indicate that Sal B prevents H_2_O_2_-induced rCMECs apoptosis, at least in part, by altering PI3K/Akt/Raf/MEK/ERK activation.

## Discussion

This study yielded four major findings: (1) Exposure rCMECs to H_2_O_2_ caused dose-dependent apoptosis, which could be prevented by pretreatment with Sal B. (2) Activation of the MEK/ERK pathway acted as a pro-apoptotic signal in H_2_O_2_-treated rCMECs; this activation was in turn dependent on PI3K activation. (3) The PI3K/Akt pathway acted as a survival signal upstream of c-Raf in H_2_O_2_-treated rCMECs. (4) Sal B exerted its preventive effects at least partly through the PI3K/Akt/Raf/MEK/ERK pathway.

CMECs is a useful cell culture model for elucidating mechanisms of cerebral vascular diseases and protection that are extremely difficult to identify *in vivo* [28]. Apoptosis of CMECs plays a pivotal role in pathogenesis of these diseases. Accumulating evidence indicates that the elevated release of ROS from brain tissue under pathologic conditions is a fundamental mechanism leading to the apoptosis of CMECs [6]. So protection of CMECs from ROS-induced apoptosis may provide beneficial therapeutic intervention to successfully combat cerebrovascular diseases. In this study, we demonstrated that Sal B was capable of saving rCMECs from apoptotic cell death caused by H_2_O_2_. This suggests that Sal B may have therapeutic use in the prevention of cerebrovascular diseases.

To date, very little is known about apoptotic effects of H_2_O_2_ in CMECs. Our results indicate that H_2_O_2_ induced CMECs apoptosis in a dose-dependent manner; this apoptosis was characterized by condensation of the nucleus chromatin, fragmentation of the DNA, and activation of caspases-3 and -9. Caspases, a family of specific cysteine proteases, are critical mediators of apoptosis. Fourteen members of the caspase family have been identified [29]. Among them, caspase-3 is a primary executioner of apoptosis induced by a variety of stimuli including H_2_O_2_ [30], [31]. Caspase-9 is a major activator in intrinsic pathway. Following cerebral ischemia, cytochrome *c* is released from mitochondrial intermembrane space as a result of the changed mitochondrion permeability [32]. Released cytochrome *c* promotes the activation of caspase-9 through Apaf-1 [33]. Activated caspase-9 subsequently activates caspase-3, which will in turn activate procaspase-9; this sequence forms positive feedback activation pathway. We showed that Sal B attenuated the activation of both caspases-3 and -9, and shortened their activation durations. The mechanisms by which H_2_O_2_ induces caspase activation in endothelial cells are not fully understood. This activation could be due to direct oxidative stress, or it could be mediated by mitochondria or by other mechanisms; any of these mechanisms might be inhibited by Sal B. Although H_2_O_2_ activated caspases in rCMECs, our data indicate that H_2_O_2_-induced apoptosis was mainly dependent on caspase-independent mechanisms but not caspase activation.

Exposure of endothelial cells to H_2_O_2_ activates several intricate cell signaling cascades that are crucial for determining whether a cell survives or dies. One such cascade involves ERK-mediated signaling [34]. The ERK pathway is most frequently associated with regulation of cell growth, survival, and differentiation [35]. A growing body of studies has revealed that ERK might play a role in apoptosis and pathogenesis. However the existing evidence is conflicting. For example, Yang et al. [7] and Wang et al. [36] reported that ERK served as pro-survival signaling mediators to alleviate H_2_O_2_ cytotoxic effects in aortic endothelial cells. Oppositely, studies by Fischer et al. [37] showed that in CMECs, paracellular permeability induced by H_2_O_2_ was due to the activation of ERK. Similarly, we showed that inhibition of ERK by U0126 elicited cell survival, suggesting ERK was a pro-apoptosis signal mediator in H_2_O_2_-stimulated rCMECs. Taken together, these results suggest that the role of ERK under oxidative stress is cell-type specific. Our data further showed that ERK activation following oxidative injury was suppressed by Sal B treatment, which was consistent with data recently published by others in different cell culture models: human aortic smooth muscle cells [38], [39]; hepatic stellate cells [40]; and human umbilical vein endothelial cells [41]. These results indicate that the ERK pathway may be a target of Sal B activity.

To gain further insight into the mechanisms by which Sal B modulates ERK signalling and by which ERK mediates H_2_O_2_-induced apoptosis, we evaluated the role of the PI3K/Akt pathway. Akt is a serine/threonine kinase. It can be activated by phosphorylation and subsequently activates multiple downstream targets to enhance cell survival. PI3K, a lipid kinase, is largely responsible for Akt phosphorylation; it has three classes or subfamilies; I, II, and III [42], [43]. Each class of PI3K has unique preferences for phosphoinositide substrates and produces specific lipid second messengers [43]. In endothelial cells, PI3K/Akt elicits a survival signalling following various stresses, including exposure to H_2_O_2_, and this signaling leads to the inhibition of apoptosis [44], [45]. Notably, following cerebral ischemia, Akt is responsible for the preventive effects on cerebrovascular endothelium apoptosis [28]. In rCMECs, we demonstrated that exposure to H_2_O_2_ induced a transient activation of Akt, which peaked at 1 h. If PI3K/Akt plays an important survival role in rCMECs, inhibition of PI3K/Akt should potentiate H_2_O_2_-induced apoptosis; indeed this was observed. Given that activation of Akt was observed in the presence of significantly elevated levels of phosphorylated ERK in cells exposed to H_2_O_2_, we were curious as to whether Akt and ERK represented two independent pathways in apoptotic signaling cascades induced by H_2_O_2_. Zhuang et al. [22] and Sinha et al. [46] recently reported that ERK was an upstream effector of Akt and that inhibition of ERK enhanced Akt activity. Unlike their observation, our data showed that Akt phophorylation level was unaffected by ERK inhibition. In contrast, H_2_O_2_-induced activation of ERK was completely inhibited by the PI3K-inhibitor, LY294002, suggesting that PI3K was responsible for ERK activation. Thus, the activation of PI3K was an upstream event in H_2_O_2_-induced rCMECs apoptosis; it subsequently activated Akt and, through an unknown mechanism, ERK. Since LY294002 inhibits all classes of PI3Ks, activation of ERK and Akt might be induced by a different PI3K family member. It was recently reported that, although all class I PI3K family members are capable of activating Akt, only PI3Kγ is responsible for the activation of MEK/ERK [47].

Clearly, although both PI3K/Akt and PI3K/ERK are activated following oxidant injury in rCMECs, they play opposite roles. PI3K/ERK signaling played an indispensably proapoptotic role in H_2_O_2_-induced rCMECs apoptosis. Sequentially activation of PI3K and Akt acted as survival signal to protect cells from apoptosis by deactivating c-Raf at Ser-259. In addition to this, Akt also promotes cell survival by its abilities to phosphorylate Bad at Ser136 [48]; Akt also directly inhibits activation of caspase-9 by phosphorylating pro-caspase-9 at Ser-196 and by this inhibits proteolytic processing of pro-Caspase-9 [49].

We suppose that the status of rCMECs apoptosis is determined by the balance between the PI3K/Akt and PI3K/MEK/ERK pathways. In the presence of H_2_O_2_, the effects of PI3K/MEK/ERK overwhelm those of PI3K/Akt, so that the balance is tipped in favor of apoptosis. So Sal B may protect rCMECs from H_2_O_2_-induced apoptosis by restoring the PI3K/Akt and PI3K/MEK/ERK balance. Our hypothesis is supported by our findings that Sal B alone triggered a rapid activation of Akt, peaked at 15 min, which then initiated downstream signaling events including deactivation of c-Raf, and down-regulation of MEK and ERK. On the other hand, inhibition of PI3K completely blocked Sal B-mediated Akt activation and all following effects. These data confirmed that PI3K/Akt is a particularly important signaling pathway in the mechanism by which Sal B promotes endothelium survival.

With a dosage that completely suppressed ERK activation, U0126 showed a substantial but not complete effect on H_2_O_2_-induced apoptosis (Fig. 2A). This clearly indicated that MEK/ERK was not the sole pathway responsible for H_2_O_2_-induced apoptosis. Using both U0126 and Sal B, we then observed a complete rescue from apoptotic cell death caused by H_2_O_2_. Thus, it appears that in addition to the PI3K/Akt/Raf/MEK/ERK pathway, Sal B might protect rCMECs from apoptosis through other mechanism(s). This possibility needs further investigation.

**Figure 4:**
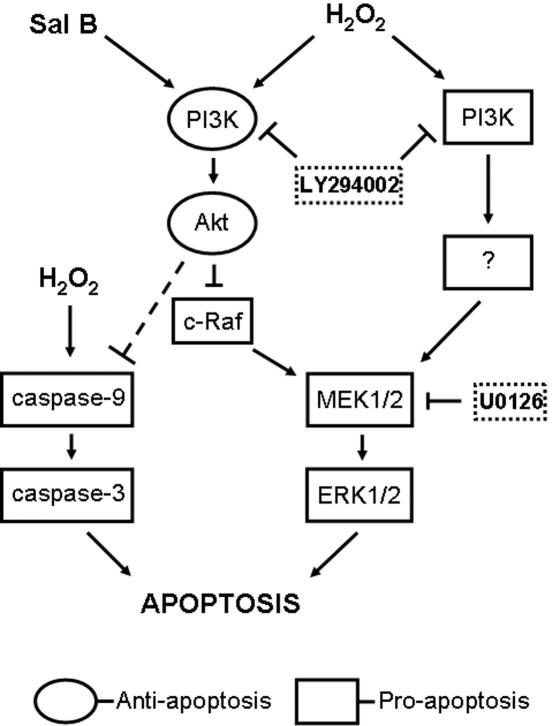
Schematic model of signaling events involved in H_2_O_2_-induced rCMECs apoptosis and Sal B preventive mechanism. The broken line indicates a possible link of Akt and caspase-9.

In conclusion, our findings have potentially important implications for understanding the mechanisms by which H_2_O_2_ induces rCMECs apoptosis and by which Sal B helps prevent apoptotic cell death (Fig. 4). To the best of our knowledge, this is the first report indicating the significance of PI3K/Akt and MEK/ERK signaling in H_2_O_2_-induced CMECs apoptosis and providing evidence that the PI3K signaling pathway is the mechanism by which Sal B acts as an anti-apoptotic agent in protecting cells from H_2_O_2_ injury and prolonging cerebral endothelial survival.

## Materials and Methods

### Reagents

Salvianolic acid B (Sal B, purity>99%) was purchased from the Chinese National Institute for the Control of Pharmaceutical and Biological Products (Beijing, China). When used, it was freshly prepared in phosphate buffer solution (PBS). Dulbecco's modified Eagle's medium (DMEM), medium 199 (M199), fetal bovine serum (FBS), PBS, Trypsin, EDTA, HEPES, PMSF, penicillin, and streptomycin were purchased from Gibco BRL (Grand Island, NY, USA). Endothelial cell growth factor (ECGF) was from Roche Diagnostics (Mannheim, Germany). U0126, LY294002 and antibodies for phospho-ERK1/2, phospho-c-Raf (Ser-259), phosphor-Akt (Ser-473), caspase-3 and caspase-9 were obtained from Cell Signaling Technology (Beverly, MA). U0124 was from CalBiochem (San Diego, CA). Horseradish peroxidase (HRP)-conjugated secondary goat anti-mouse or anti-rabbit antibodies were from Invitrogen (S. San Francisco, USA); ECL reagent kit was from Pierce Biotechnology (Rockford, USA); Heparin, collagenase II, gelatin, H_2_O_2_, zVAD-fmk, GW5074, and antibodies for β-actin and γ-tubulin were purchased from Sigma (St. Louis, MO, USA). H_2_O_2_ was freshly prepared for each experiment from a 33% stock solution.

### Cell culture and drug treatments

Rat cerebral microvascular endothelial cells (rCMECs) were isolated from Sprague-Dawley rat cerebral cortex microvessel segments, according to the method described by Bederson et al. [50]. Briefly, the cortices were dissected free of meninges and white matter in M199 supplemented with 8% FBS, 10 U/ml heparin and 100 U/ml penicillin-streptomycin solution. The remaining gray matter was cut into small pieces and homogenized. Thereafter, the slurry was filtered consecutively through 145- and 75-µm nylon mesh screens to remove large vessels, tissue mass, single blood and nerve cells. The collected cerebral microvessels were treated with 0.1% collagenase at 37°C for 15 min. After incubation, the detached cells were centrifuged and resuspended in DMEM supplemented with 25% FBS, 10 U/ml heparin, 100 U/ml penicillin-streptomycin solution and 150 µg/ml ECGF, and were grown in monolayers at 37°C in a humidified atmosphere of 5% CO_2_ and 95% air. Cells from 4^th^ and 7^th^ passage were used in this study. For all experiments, rCMECs were grown to 80%–90% confluence and then pretreated with designated agents for 60 min prior to H_2_O_2 _exposure in fresh medium.

### TUNEL assay

H_2_O_2_-induced apoptosis was detected by performing the terminal deoxynucleotidedyl transferase-mediated dUTP nick end-labeling (TUNEL) assay using an Apo-Direct™ Kit (CalBiochem, San Diego, CA). TUNEL was performed according to the manufacturer's instructions. Briefly, after pretreatments and exposure to H_2_O_2_, cells were harvested, washed, fixed, permeabilized, and labeled for DNA strand breaks, then analyzed on a Coulter Epics Elite flow cytometer (Beckman-Coulter, Miami, USA). All assays were carried out in triplicate.

### Western Blot

Protein extracts were prepared and subjected to Western blot analysis as described by Sambrook et al [51]. In brief, after designated treatment, endothelial cells were scraped off the plates, washed with PBS and dispersed in 5 volumes of ice-cold suspension buffer (100 mM NaCl, 10 mM Tris-Cl, 1 mM EDTA, and 100 µ/mL phenylmethylsulfonyl fluoride). An equal volume of 2× SDS gel-loading buffer (100 mM Tris-Cl, pH 6.8, 200 mM DTT, 4% SDS, 0.2% bromphenol blue, and 20% glycerol) was added, and the samples were boiled for 10 min. After centrifugation, protein extracts were resolved on SDS-polyacrylamide gel electrophoresis (PAGE) and transferred onto polyvinylidine difluoride membranes. After blocking with 5% skim milk in TBS-T (150 mM NaCl, 50 mM Tris-HCl, pH 8.0, and 0.1% Tween 20) for 1 h, the membranes were probed with various first and second antibodies and developed with enhanced chemiluminescence by following the manufacturer's instructions (Pierce). The levels of proteins were determined using densitometry with Image J software, which allowed direct comparisons between experimental sets.

### Statistical analysis

Data was analyzed with unpaired two-tailed Student's *t*-test or one-way ANOVA followed by Tukey's multiple comparison test with GraphPad Prism software (San Diego, CA). Data were expressed as mean±SEM derived from at least three independent experiments. Differences were considered significant at *P*<0.05.
